# Histopathologic and Radiologic Assessment of Chemotherapeutic Response in Ewing's Sarcoma: A Review

**DOI:** 10.1155/2012/357424

**Published:** 2012-03-01

**Authors:** José M. García-Castellano, Nagib Atallah Yordi, Carolina Reyes, John H. Healey

**Affiliations:** ^1^Research Unit, Hospital Universitario de Gran Canaria Dr. Negrín, 35010 Las Palmas de Gran Canaria, Spain; ^2^Orthopaedic Service, Complejo Hospitalario Universitario Insular-Materno Infantil, 35016 Las Palmas de Gran Canaria, Spain; ^3^Department of Arthroscopic and Minimally Invasive Surgery, Al Noor Hospital, Abu Dhabi, UAE; ^4^Department of Pathology, Memorial Sloan-Kettering Cancer Center, New York, NY 10065, USA; ^5^Orthopaedic Service, Department of Surgery, Memorial Sloan-Kettering Cancer Center, Weill Cornell Medical College, 1275 York Avenue, New York, NY 10065, USA

## Abstract

Ewing's sarcoma is a highly malignant tumor that metastasizes rapidly and is thus associated with a low survival rate. The intensification of chemotherapy has been shown to improve the overall survival of patients with Ewing's sarcoma. However, intensified chemotherapy can lead to increased toxicity or even the development of secondary malignancies. The stratification of patients with Ewing's sarcoma into “good” and “poor” responders may help guide the administration of progressively more intensified chemotherapy. Thus, an accurate assessment of the chemotherapeutic response, as well as the extent of chemotherapy-induced tumor necrosis, is critical for avoiding potential treatment-related complications in these patients. This paper reviews the methods currently used to evaluate chemotherapeutic response in Ewing's sarcoma, focusing specifically on histopathologic and imaging analyses, and discusses novel therapies and imaging methods that may help improve the overall survival of these patients.

## 1. Introduction

The dramatic improvement in the survival of patients with Ewing's sarcoma during the past 2 decades can be attributed to the use of aggressive chemotherapy. In the absence of chemotherapy, this highly malignant tumor quickly metastasizes, even when adequate local control has been achieved. In patients without evidence of metastatic disease at presentation, treatment protocols for Ewing's sarcoma incorporating chemotherapy, surgery, and radiotherapy result in 5-year disease-free survival rates of 40%–50% [1, 2]. The inclusion of intensified chemotherapy improves the 2-year event-free survival rate to 77% [3]. This finding suggests that further intensification of chemotherapy may improve local disease control; however, this can potentially lead to the onset of additional toxic effects and even to the development of secondary malignancies. 

 Defining prognostic variables may finally permit the stratification of patients into “poor-risk” and “good-risk” subgroups. This would allow the administration of progressively more intensified therapy in the poor-risk subgroup, decreasing the probability of choosing drug-resistant cellular clones, with an increased risk of metastasis [4]. In the good-risk subgroup, less intense and therefore potentially less-toxic therapy may be sufficient.

The ability to predict the survival of patients with Ewing's sarcoma is limited, both at the time of diagnosis and after initial preoperative chemotherapy. Clinical signs are insufficient for determining the effectiveness of preoperative chemotherapy and are poorly correlated with histologic tumor response [5]. The strongest predictor of outcome is the presence or absence of metastatic disease [2, 3]. But this prognostic indicator is not useful in most patients with Ewing's sarcoma who present with apparently localized disease. In these patients, 2 intrinsic factors and 1 treatment response factor have been identified that predict outcome. Tumor size and genetic profile are strong predictors of outcome. For example, tumors with genetic alterations in p53 or p16/p14^ARF^ have more aggressive behavior and a worse response to chemotherapy [6]. However, these prognostic factors are not under the physician's control. In contrast, the histologic response of the primary tumor to preoperative chemotherapy is a powerful predictor of the occurrence of relapse, which may be influenced by the treatment team [7, 8].

In this paper, the methods designed to assess chemotherapy-induced tumor necrosis and how this parameter can influence the prognosis and treatment of Ewing's sarcoma will be reviewed.

## 2. Histopathologic Assessment

Resection of the primary tumor is the best option for reducing the bulk of soft tissue tumors and has the potential to eliminate tumor cells entirely from bone and soft tissue [9]. Thus, resection eliminates disease that could cause local recurrence and further metastasis [10]. Although amputation is appropriate for certain cases, limb preservation is possible in most cases. Approximately 50% of Ewing's sarcoma cases overall, as well as 70% of extremity cases, are surgically resectable. The histologic evaluation of resected specimens allows the effectiveness of preoperative chemotherapy to be accurately evaluated [11, 12].

**Figure 1:**
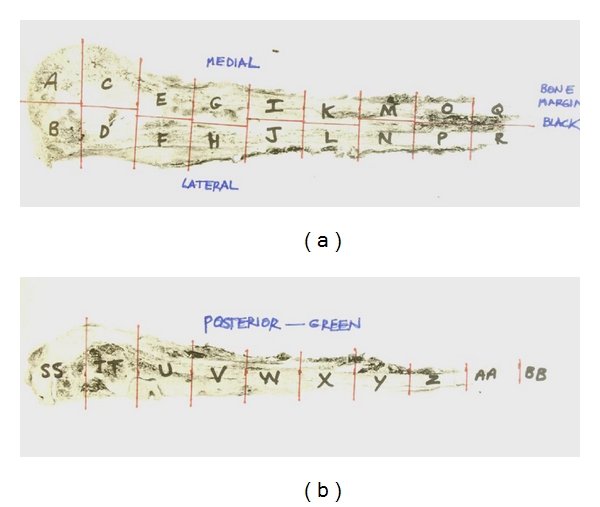
Fibrotic replacement of the marrow space occurred following chemotherapy. There were small foci of residual disease grossly and histologically. Overall, systematic mapping of the resection specimen is essential to measure the response to preoperative chemotherapy in the primary tumor (a). Anteroposterior view of the specimen mapped into block segments (b). This response was graded as good, with more than 90% necrosis (Grade 3, Huvos system; Grade 2, Picci system).

Essentially, 2 different methods of histologic assessment have been established. The first, described by Huvos, is based on his method for evaluating osteogenic sarcoma samples [13]. In a semiquantitative manner, histologic evaluation is performed by grading the extent of necrosis relative to the percentage of residual viable tumor. The Huvos system includes 4 grades: Grade 1: little or no evidence of necrosis; Grade 2: necrosis of 50%–90%; Grade 3: necrosis between 90%–99%; finally, Grade 4: 100% necrosis (Figure 1) [7].

This method of histologic grading has been shown to be very effective in the management of Ewing's sarcoma. The extent of necrosis has been directly correlated with improved survival [7]. However, quantitative measurements are open to criticism. Ewing's sarcoma differs from osteogenic sarcoma in that it does not produce any major extracellular matrix component, so there is no indicative evidence left by the tumor cells. Furthermore, in response to chemotherapy, Ewing's sarcoma cells may disappear completely. For these reasons, there may be a dramatic decrease in tumor volume after preoperative chemotherapy without histologic delineation of where the tumor was located originally [14]. Because of this potentially large change in tumor volume following neoadjuvant chemotherapy, estimates of tumor necrosis are difficult to calculate based only on the viable cells per unit area of residual tumor. Consequently, a strictly quantitative method to estimate tumor necrosis may not be appropriate in Ewing's sarcoma [15]. However, because pathologists are accustomed to using the well-established Huvos system for osteogenic sarcoma, they are able to apply it accurately to Ewing's sarcoma.

Instead of estimating the amount of nonviable tumor, Picci et al. [15] proposed evaluating the amount of remaining viable tumor. They developed a method that requires calculating the absolute quantity of viable tumor cells after preoperative chemotherapy, which does not vary with volume changes of the primary tumor. The scoring system proposed by Picci et al. [15] includes 3 grades. Grade 1 response represents a tumor with at least 1 macroscopic residual nodule of viable tumor. Individual macroscopic nodules are defined as those that are larger than one 10x magnification field, or as scattered microscopic nodules that individually are smaller than one 10x magnification field but that collectively are larger than one 10x magnification field. Grade 2 response represents a tumor with only isolated microscopic foci of viable tumor smaller than the size of a 10x magnification field. Grade 3 response indicates no evidence of viable tumor cells [14]. This method is easy to interpret because it does not require the calculation of percentages, but it does require an exhaustive examination of the tissue and preparation of multiple sections [8]. Furthermore, this method fails to account for the original tumor size. For example, the persistence of 1 nodule is graded the same, regardless of whether the tumor volume was 10 cm^3^ or 200 cm^3^.

**Figure 2:**
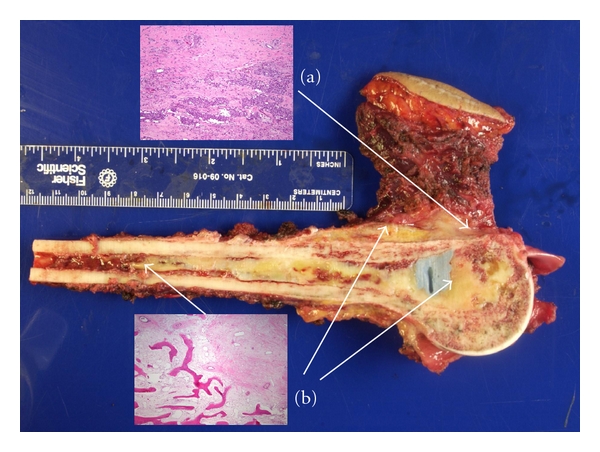
Gross specimen and whole-mount sections demonstrating the areas of (a) necrosis and (b) residual disease.

Akermån [16], who graded specimens of Ewing's sarcoma using the 2 scoring systems described above, showed similar disease-free survival. Akermån stressed that the regional mapping protocol was more important than the type of grading system used (Figure 2). Picci et al. [15] support this point by showing the significance of this regional mapping of the tumor. They found that viable tumor was more often detected in some of the preferential or sanctuary sites than in the central part of the tumor. These preferential sites were: (1) in the subperiosteal region of new bone formation, (2) in the soft tissue mass, and (3) in areas of hemorrhage, present in 41% of patients. Furthermore, disease was present in the intramedullary canal in 36% of patients.

Thus, an acceptable histologic response grading system depends on a meticulous and precise macroscopic and microscopic examination of the surgical specimen. To accomplish this aim, it is very important to observe the following steps. First, it is necessary to examine the fresh specimen very soon after surgery. Second, it is crucial to keep multiple sections from the preferential sites. Third, it is imperative to cut various sections from the area where the biopsy was obtained. Fourth, it is useful to saw the specimen into halves, using one half for the multiple sections and the other half as a whole tumor section. Fifth, the places where the cuts were made must be represented in an illustration of the specimen and kept as part of the permanent record.

## 3. Imaging Analysis

Precise imaging methods have allowed the noninvasive identification, localization, and quantification of residual viable tumor during and after preoperative chemotherapy in patients with Ewing's sarcoma. Diagnostic imaging may also influence the adjustment of neoadjuvant chemotherapy schedules or the timing of surgical intervention [11, 17]. However, while estimates of tumor changes based on diagnostic imaging studies are more reliable than those based on clinical methods, they do not always predict histopathologic response [12]. More refined imaging techniques are needed to monitor tumor status during treatment and to predict tumor response to treatment [18].

### 3.1. Conventional Radiography

Conventional radiography is still useful for developing differential diagnoses, detecting pathologic fractures, estimating tumor aggressiveness, and during followup [19]. However, radiographs cannot be used to accurately indicate the extent of medullar involvement or to delineate soft tissue masses, unless the masses are heavily calcified. In addition, reductions in tumor size may be underestimated or overestimated on plain film [11].

### 3.2. Radionuclide Studies

Semiquantitative analysis of tumor activity can be achieved by means of radionuclides, because uptake of the labeled compound depends on cellular function.

 Static studies with methylene diphosphonate (MDP) labeled with technetium-99m (^99m^Tc) are not useful for evaluating primary tumors because they often exaggerate the extent of the tumor. However, serial scintigrams have been used to measure activity in the tumor, which is compared with activity in normal contralateral bone. These dynamic studies of ^99m^Tc-MDP have been utilized to distinguish between good responses and poor responses to chemotherapy [20].

Uptake of gallium-67 (^67^Ga)-citrate more closely defines the actual tumor than that of ^99m^Tc-MDP because ^67^Ga-citrate is taken up quickly by Ewing's sarcoma cells [21]. When findings on a bone scintigram with ^99m^Tc-MDP remain abnormal and no pathologic fracture is present, normal findings on a gallium study suggest that no residual malignant disease is present [22].

Because of the rapid clearance from the blood and the lack of accumulation in nonneoplastic bone of thallium-201 (^201^Tl), ^201^Tl scans appear to be more accurate than ^67^Ga-citrate scans or bone scans with ^99m^Tc-MDP in indicating the course of disease. However, ^201^Tl scintigraphy has not been widely used for this purpose [23, 24].

### 3.3. Computerized Tomography (CT)

 Computerized tomography (CT) provides a cross-sectional view of sarcoma of long bones. Its contrast resolution permits visualization of the extraosseous soft tissue mass and involved bone marrow [11]. However, in chemoresponsive tumors, CT changes in the affected bone marrow do not differentiate active tumor from intramedullary necrosis [25]. The wide availability and relative low cost of CT are attractive, but its sensitivity is probably not sufficient, even when used with intravenous contrast. CT may identify a persistent soft tissue mass after induction chemotherapy. This correlates with a residual soft tissue extension of tumor, incomplete response to chemotherapy, and diminished survival in these patients compared with patients without persistent disease extension beyond the bony compartment [26].

## 4. Magnetic Resonance Imaging

### 4.1. Conventional Static Magnetic Resonance Imaging (MRI)

Efforts to correlate modifications in static magnetic resonance (MR) signal intensity with therapeutic response have yielded conflicting results. Discrimination of good and poor responders by means of conventional static MR imaging (MRI) is mainly based on subjectively interpreted qualitative parameters [11, 27]. Moreover, marked overlap has been reported between responders and nonresponders using this technique [27]. For example, using static MRI, Erlemann et al. [12] found a decrease in tumor volume after chemotherapy in 73% of responders and in 50% of nonresponders, which means an accuracy of 61.9%. They found that this decrease in tumor volume was not a significant indicator of response (*P* > .05).

Some limitations in the use of conventional static MRI have been observed. Holscher et al. [27], on T2-weighted MR images, found a complete concordance between changes in the signal intensity of the extraosseous tumor component, modifications in tumor volume, and histopathologic findings in 70% of patients. They concluded that changes in the signal intensity of the extraosseous tumor component could be indicative of a response to chemotherapy. However, no correlation was found between changes in the intraosseous tumor component and modifications in tumor volume or histopathology. The results of Erlemann et al. [12] contradict those of Holscher et al. [27], who found a correlation between a decrease in T2-weighted signal intensity of the soft tissue mass and a favorable histologic response after preoperative chemotherapy. However, it is known that peritumoral, or paratumoral, edema, which appears on T2-weighted MR images as a border of high signal intensity surrounding the tumor margins [28], may also decrease in response to chemotherapy. Hence, these changes are not very specific.

The interpretation of signal intensity on T2-weighted MR images remains a problem. In general, low signal intensities are related to acellular tissues, whereas high signal intensities represent the more cellular parts of the tumor [29]. However, MacVicar et al. [9] found microscopic clusters of viable cells in areas of both high- and low-signal intensity after treatment in 10 patients (70%). Moreover, they found patients that still had microscopic tumor clusters even though all the soft tissue tumors were so considerably reduced in size that signal evaluation was impossible. This could be the reason that Van Der Woude et al. [30] could not identify minimal residual disease (defined as <10% of entire tumor volume) in specimens in which residual foci of viable cells were observed histologically. This means that MRI cannot exclude the presence of residual disease activity. So, although a pattern of modification in signal intensity is qualitative evidence of a chemotherapeutic effect, it is questionable for excluding active disease [31].

### 4.2. Dynamic MRI

 Dynamic MR studies with injection of gadopentetate dimeglumine have been used to improve MR images for assessing response to chemotherapy [12]. Dynamic contrast-enhanced MRI is useful for detecting the most viable parts of the tumor and serves as an initial pattern for followup of the tumor treatment. Combined with histopathologic assessment, dynamic imaging parameters are recommended for evaluating the effect of neoadjuvant chemotherapy in patients with Ewing's sarcoma [32]. Thus, dynamic contrast-enhanced MRI correlates with percent necrosis as determined by pathologists. This noninvasive method is a useful tool for surgical planning [33].

In this technique, tumor signal intensity is drawn from serial images obtained at 15- to 20-s intervals, and the inclination of the resultant time–intensity curve is calculated. Different patterns have been described.

Brisk slopes (>30%) represent higher perfusion or faster uptake of the contrast agent, perhaps due to tumor neovascularization. These profiles suggest the presence of viable tumor [17, 34]. On dynamic MRI, tumor foci as small as 3–5 mm^2^ can be detected. These foci are not an exceptional finding in Ewing's sarcoma after chemotherapy [35], and, as Picci et al. [8] showed, they are also important for prognosis. Nevertheless, dispersed or smaller-dimension nests of viable tumor cells cannot be distinguished with dynamic contrast-enhanced MRI. Furthermore, complete absence of early enhancement does not exclude the presence of disseminated viable cells [17].

Late and gradually enhancing or nonenhancing areas correspond histopathologically to regions of chemotherapy-induced necrosis, mucomyxoid degeneration, or fibrosis. Alternatively, this response is associated with reactive alterations such as edema, hemorrhage, or osteomyelitis, or with tumor-related extracellular matrices such as abundant osteoid or chondroid.

Early and continuously amplified structures seen on MRI correspond to tumor-feeding arteries, growth plate vessels, or remnant viable tumor at specific sites.

In general, responsive tumors show more gradual increases of gadopentetate dimeglumine after preoperative chemotherapy than do nonresponsive tumors. But retarded uptake has been observed in necrotic areas, in cystic regions, and in cartilaginous or myxomatous tissue [36].

Comparing the accuracy of different imaging techniques in evaluating the response to preoperative chemotherapy in Ewing's sarcoma, Erlemann et al. [12] assessed chemotherapeutic response with MRI, both with and without gadolinium diethylene-triamine-pentaacetic acid (Gd-DTPA) enhancement, and with dynamic Gd-DTPA studies, and the results were compared with those of skeletal scintigraphy. Of all the techniques employed, dynamic MRI had the highest degree of accuracy (85.7%) and was superior to scintigraphy, particularly in patients who were receiving intraarterial chemotherapy [12]. Compared with skeletal scintigraphy, dynamic MRI has a clearly superior spatial resolution, and areas of predilection for the persistence of tumor cells can be examined directly. Compared with angiography, which also has a high spatial resolution, dynamic MRI is less invasive. Although this method has produced promising results, it requires relatively complex manipulation of quantitative data and is currently unlikely to be adopted as a routine radiologic practice [9].

Dynamic MRI does have some limitations, as it has been observed to yield some false-positive results. The large pathologic vessels in a zone of active subperiosteal new bone formation, and the physeal vessels in young patients, occasionally lead to overestimation of tumor extent, especially towards the growth plate [17].

## 5. Tumor Vascularization Assessment

Because Ewing's sarcoma commonly is an extremely vascular tumor and because tumor neovascularization is associated with prognosis and response to therapy in different human neoplasms [37], changes in tumor neovascularization can be analyzed to evaluate the result of preoperative chemotherapy [38, 39]. It has been reported that rapid disappearance of tumor vessels is related to a favorable response to chemotherapy, while permanent pathologic vascularity implies a poor response [40]. Several techniques have been designed for assessing tumor vascularization.

### 5.1. Magnetic Resonance Angiography (MRA)

Magnetic resonance angiography (MRA) permits the study of tumor neovascularity in vivo [37]. This tumor characteristic appears to correlate with tumor aggressiveness and the presence or absence of metastases [38]. In patients who responded to chemotherapy, MRA showed a marked reduction in tumor neovascularity, whereas in patients who did not respond to chemotherapy, MRA demonstrated persistent or increased tumor neovascularity [37].

### 5.2. Color Doppler Flow Imaging (CDFI)

Color Doppler flow imaging (CDFI) has also been used to estimate the response to preoperative chemotherapy in patients with Ewing's sarcoma. Parameters used in this technique are related to the modification of blood flow resistance. The disordered structure of the vascularity of viable tumor reduces the resistance of the peripheral vascular bed. This is the main reason that the peripheral resistance of tumor-feeding arteries is decreased or unaltered. Additionally, a persistent intratumoral flow is found. These 2 parameters suggest a poor histologic response to chemotherapy in Ewing's sarcoma [41]. In contrast, an increased resistive index is indicative of a good response [39].

With CDFI it is possible to obtain qualitative as well as quantitative parameters with spectral analysis. In this way, estimation of qualitative anomalous flow patterns within tumors, and quantitative evaluation of tumor blood flow supply and intratumoral blood flow have been performed [42].

In monitoring the effect of chemotherapy in Ewing's sarcoma, CDFI with spectral analysis has some advantages over dynamic gadolinium-enhanced MRI and 3-phase bone scintigraphy because of its claimed superior accuracy, noninvasive nature, accessibility, short duration examination, and low cost [39, 42].

However, CDFI also has some disadvantages. It is technically difficult to perform; its reproducibility needs to be proven; it has poor spatial resolution; and it is not useful for determining chemotherapeutic response in purely intraosseous tumors [42]. CDFI is also deficient when there is a concomitant healing fracture or a significant hypoxic area around the tumor [39].

## 6. Novel Imaging Techniques

Preliminary results using MR spectroscopy have demonstrated its ability to show some metabolic modifications in chemoresponsive tumors. However, these results have not been proven in the clinical setting. Positron emission tomography (PET) is another imaging technique under consideration for assessing the effectiveness of neoadjuvant chemotherapy in Ewing's sarcoma [11].

Imaging techniques such as CT or MRI cannot distinguish accurately between active and necrotic tumor cells. Furthermore, these techniques are limited in their ability to discriminate viable tumor cells from posttherapeutic changes or to exclude minimal residual disease [43, 44].

PET is increasingly being used as a diagnostic technique. Because of the similarity between 2-[fluorine-18]fluoro-2-deoxy-d-glucose (FDG) and glucose, PET can be used to detect malignancies with glucose hypermetabolism [43, 45].

While conventional imaging modalities use morphologic criteria to differentiate between benign and malignant tumors, FDG PET utilizes an increased demand for glucose, which is proportional to FDG uptake [44].

In several malignancies, PET can accurately predict pathologic changes, differentiate between local and disseminated disease, evaluate the response to therapy, and detect relapsed tumors [46, 47].

In patients with Ewing's sarcoma, FDG PET correlates with histologic response to neoadjuvant chemotherapy [5], with a sensitivity and specificity of about 96% and 78%, respectively [48].

However, PET cannot identify the precise anatomic localization of lesions because of its limited spatial resolution. But the combination of PET with CT mitigates this limitation [43].

In addition, PET/CT is more accurate than PET alone for patients with Ewing's tumors [43], because CT acquires the anatomic data while PET obtains the metabolic information [45].

## 7. Influence of Necrosis Assessment on Prognosis and Treatment

### 7.1. Prognosis

The parameters obtained from the different methods of assessment described above have important implications for prognosis. A strong correlation between prognosis and tumor volume and necrosis has been observed in patients treated with preoperative chemotherapy and surgery [8, 49]. When there is an increase in tumor size after chemotherapy, the histopathologic evaluation shows an inadequate response, whereas when there is a decrease in tumor size, the histopathologic evaluation shows a good response [30]. Thus, the inadequate response to chemotherapy in large tumors is associated with the presence or development of drug-resistant clones [5].

The risk of local recurrence and metastatic disease are most strongly associated with the status of operative margins [7]. In 1 study, an inadequate operative margin was the only factor that influenced the risk of local recurrence [50]. An important association between margin status and the effectiveness of preoperative chemotherapy has been reported by other investigators [7]. The probability of local recurrence of Ewing's sarcoma persists even in patients who have negative resection margins, who have a good histologic response to chemotherapy, and who receive local radiotherapy [51].

### 7.2. Treatment

 The classification of patients into good responders and poor responders through the careful assessment of necrosis may encourage the development of new treatment strategies. In particular, poor responders would be treated with more aggressive therapy.

Preoperative chemotherapy has become one of the cornerstones in the treatment of patients with Ewing's sarcoma [18]. Preoperative chemotherapy has some advantages; namely, it can be used to treat the disease early, diminishing the likelihood of metastatic dissemination, or to reduce tumor volume, permitting complete tumor resection without the need for limb amputation.

Different treatment protocols have been used in Ewing's sarcoma. In those patients without evidence of metastatic disease at presentation, the combined treatment with chemotherapy, surgery, and radiotherapy produces a 5-year disease-free survival rate of 40%–50% [1, 2].

The addition of ifosfamide and etoposide to the standard chemotherapy regimen of vincristine, dactinomycin, cyclophosphamide, and doxorubicin (VACA + IE) has been shown to improve survival. Since the incorporation of these 2 drugs, the disease-free survival rates have increased to between 62% and 78% [2, 52–54]. In terms of histologic response, these 2 agents have produced significantly better results, especially when ifosfamide is employed early in the treatment [54].

New treatments using alkylating agents, at an even higher dose intensity, have produced a 2-year event-free survival rate of 77% [3]. Other investigators, however, have not found an improvement in the outcome of patients using this treatment approach [55]. Moreover, recent reports have revealed a disturbing rate of secondary acute myelogenous leukemia although, as shown by Bacci et al. [56], this increased risk may be influenced by the use of concomitant radiotherapy.

In terms of surgical technique, more precise histologic and radiologic techniques have allowed better demarcation of the operative margins and have helped in the evaluation of residual viable tumor at specific preferential sites. In patients with a poor response to chemotherapy (Grade 1 or 2), there is a greater probability of local recurrence than in those with a good response to chemotherapy (Grade 3 or 4) (12.5% versus 4.5%) [7]. Efforts to obtain wide surgical margins must be intensified in these patients and greater consideration given to adding postoperative radiotherapy, regardless of the adequacy of the histologic margin.

Traditionally, the primary tumor has been treated with definitive local therapy using radiotherapy [57]. Local control has been improved when patients receive doses greater than 49 Gy, when the tumors are 8 cm or smaller, or when patients receive doses greater than 54 Gy for tumors larger than 8 cm [58].

Currently, most cases of Ewing's sarcoma are treated by limb salvage surgery combined with neoadjuvant chemotherapy, which achieve patient survival and preserve function [59]. When an adequate surgical margin can be achieved after preoperative chemotherapy, radiotherapy is not used. Postoperative radiotherapy is reserved for cases in which (1) the operative margin status was questionable or (2) the response to chemotherapy was poor. Preoperative radiotherapy is reserved for cases in which (1) the response to chemotherapy was limited and (2) complete surgical excision would not be possible or would require sacrificing a critical structure(s). Because of the necrosis and fibrosis caused by radiation, it is impossible to assess the response to chemotherapy if radiotherapy is administered preoperatively.

New therapies have been developed for those patients in the high-risk subgroup. These new treatment protocols use conventional chemotherapy and consolidation with very-high-dose short-term chemotherapy containing busulfan and melphalan, followed by autologous blood stem cells [3, 60]. In certain groups of high-risk patients, consolidation with myeloablative total-body irradiation and chemotherapy followed by stem cell rescue might improve prognosis [61]. However, the available literature does not reveal a clear advantage for consolidation with high-dose chemotherapy [62].

Finally, other treatments like immunotherapy [63, 64] and bisphosphonate therapy [65] have also been introduced recently in Ewing's sarcoma. Interferon (IFN) beta (IFN-*β*), and to a lesser degree IFN alpha (IFN-*α*), inhibits Ewing's tumor cell proliferation. In a nude mouse model of Ewing's tumor xenografts, human-type IFN-*α* and (IFN-*β*), demonstrated an antitumoral effect. In addition, human IFNs enhance the antitumor effect of ifosfamide. This combined synergistic treatment induces a remarkable decrease in the mitotic index and manifest necrosis [63]. Furthermore, this treatment provokes the downregulation of angiogenic factors such as vascular endothelial growth factor, matrix metalloproteinase-9, and urokinase plasminogen activator receptor [64]. On the other hand, the bisphosphonate zoledronic acid induces apoptosis and inhibits primary bone tumor growth through a mechanism involving the upregulation of osteoprotegerin in a primary Ewing's sarcoma mouse model [65].
